# A sparse-projection computed tomography reconstruction method for in vivo application of in-line phase-contrast imaging

**DOI:** 10.1186/1475-925X-12-75

**Published:** 2013-07-30

**Authors:** Liting Wang, Xueli Li, Mingshu Wu, Lu Zhang, Shuqian Luo

**Affiliations:** 1 College of Biomedical Engineering, Capital Medical University, You An Men, Beijing 100069, People’s Republic of China; 2 Department of Radiology, Guangzhou First People’s Hospital, Guangzhou 510180, People’s Republic of China

## Abstract

**Background:**

In recent years, X-ray phase-contrast imaging techniques have been extensively studied to visualize weakly absorbing objects. One of the most popular methods for phase-contrast imaging is in-line phase-contrast imaging (ILPCI). Combined with computed tomography (CT), phase-contrast CT can produce 3D volumetric images of samples. To date, the most common reconstruction method for phase-contrast X-ray CT imaging has been filtered back projection (FBP). However, because of the impact of respiration, lung slices cannot be reconstructed in vivo for a mouse using this method. Methods for reducing the radiation dose and the sampling time must also be considered.

**Methods:**

This paper proposes a novel method of in vivo mouse lung in-line phase-contrast imaging that has two primary improvements compared with recent methods: 1) using a compressed sensing (CS) theory-based CT reconstruction method for the in vivo in-line phase-contrast imaging application and 2) using the breathing phase extraction method to address the lung and rib cage movement caused by a live mouse’s breathing.

**Results:**

Experiments were performed to test the breathing phase extraction method as applied to the lung and rib cage movement of a live mouse. Results with a live mouse specimen demonstrate that our method can reconstruct images of in vivo mouse lung.

**Conclusions:**

The results demonstrate that our method could deal with vivo mouse’s breathing and movements, meanwhile, using less sampling data than FBP while maintaining the same high quality.

## Background

Lung disease has become increasingly common. Lung diseases range from the common cold to life-threatening conditions such as bacterial pneumonia, pulmonary embolisms, and lung cancer. The lungs are the essential respiration organs in many air-breathing animals. Their principal function is to transport oxygen from the atmosphere into the bloodstream and to release carbon dioxide from the bloodstream into the atmosphere. Lung tissue is a typical soft tissue that has received extensive attention from many research groups to reveal its detail structures.

X-rays [1] have been widely used to reveal the detailed structures of bodies in medical applications. However, it is difficult to image soft tissue with X-rays. Imaging the lung tissue results in a low absorption contrast because the lung’s anatomical structure has multiple air-tissue alveoli. Phase-contrast imaging (PCI) is a new imaging mechanism intended to solve the problem of soft tissue imaging. PCI is based on using X-rays’ phase change through objects to obtain a phase contrast image. For hard X-rays (10–100 keV), the X-ray absorption of soft tissues is low, but the phase change caused by soft tissue is approximately 1000 times. Therefore, PCI performs better than absorption imaging and substantially increases the X-ray contrast resolution of soft tissue imaging. Currently there are three primary types of techniques that have been used for PCI: X-ray interferometry [2], diffraction-enhanced imaging (DEI) [3] and in-line holography [4].

The in-line phase contrast X-ray imaging method was developed by Snigirev et al. at European Synchrotron Radiation Facility (ESRF) [5] and by Wilkins et al. at Commonwealth Scientific and Industrial Research Organization (CSIRO) [6]. In-line holography of PCI is a powerful phase-sensitive technique that generates high spatial resolution and super contrast of weakly absorbing objects compared with conventional radiography by using a propagation-based technique. In this method, X-rays are transmitted through the sample object at various angles and then received by the detector within a certain distance. When the X-rays propagate through the sample, the shape of the wave-front changes because the varying thickness of the sample causes differences in the X-ray refractive index, then, an edge-enhanced contrast is observed.

Phase contrast X-ray imaging methods have been applied to mouse’s lung [7-9] and propagation-based methods have been applied to mouse’s lung and exhibit a speckled intensity pattern that is attributed to the air-filled alveoli [10]. For three-dimensional observation of lung tissues using this method, phase contrast X-ray computed tomography (PCX-CT) [11] has been developed. PCX-CT was achieved by introducing the technique of X-ray phase contrast imaging into X-ray CT. It has the characteristics of high sensitivity and three-dimensional imaging of lung tissues [12]. A 3D lung model is built, and the experiment is implemented in a dead mouse. It remains challenging to implement for a live mouse. During normal breathing, expiration is passive and no muscles are contracted (the diaphragm relaxes). The rib cage itself is also able to expand and contract to some degree through the action of other respiratory and accessory respiratory muscles. Consequently, air is transported into or expelled out of the lungs. This type of lung is known as a bellows lung, and it causes difficulty in lung tissue registration and alignment at various angles.

In this paper, we present a compressed sensing-based (CS-based) optimization method for CT reconstruction and apply this method to an in vivo in-line phase contrast imaging experiment. The proposed method has two primary contributions. 1) It addresses the problems caused by a live mouse’s respiration. Respiratory phases are classified and extracted into different stages. For example, the rib cage’s two stages are expansion to maximum and contraction to minimum. 2) It addresses the sparse 3D reconstruction problem. Respiratory phase extraction causes the number of images to be reduced at each angle. A compressed sensing-based (CS-based) optimization method for CT reconstruction is applied to address this problem. This research is also important for performing CT image reconstruction from a small number of projections, which can shorten the scan time and reduce the radiation dose.

## Methods

### Optical system

The basic theory of in-line phase-contrast X-ray imaging is Fresnel diffraction. X-rays propagate through the sample object and are received by the detector within a certain distance. During the propagation, the shape of the X-ray wave front changes because of variations in the thickness and the X-ray refractive index of the sample [13].

**Figure 1:**
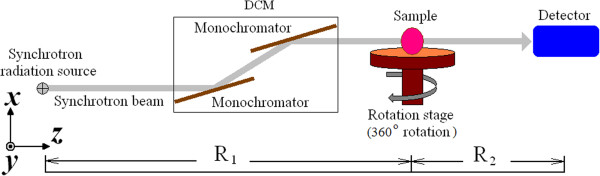
Schematic of an in-line phase contrast imaging configuration.

Figure 1 shows a schematic of an in-line phase contrast imaging configuration. In Figure 1, R_1_ is the source-to-object distance and R_2_ is the object-to-detector distance (In the experiment, R1 is set to 34 m, R2 is set to 1.2 m). To achieve phase-contrast imaging based on diffraction effects, the X-ray source should provide a sufficient degree of spatial coherence. In the X-ray region, a wave field propagating in the z direction is expressed under the paraxial approximation by

(1) $$\frac{2\pi }{\lambda }\frac{\partial }{\partial z}I\left(x,y,z\right)=\nabla_{⊥}\left[I\left(x,y,z\right)\nabla_{⊥}\Phi \left(x,y,z\right)\right],$$

which is known as the transport of intensity equation (TIE) [14,15], where ∇_⊥_ is the two-dimensional gradient operator acting in the x-y plane. In the near-field Fresnel region, TIE can address the change of the wave front caused by propagation as Φ(*x*, *y*, *z*) in eq. (1).

### In the case of weak absorption and unit-amplitude plane-wave illumination, eq. (1) is simplified to

(2) $$I\left(x,y,z\right)\approx 1+\frac{\mathrm{\lambda z}}{2\pi }\nabla_{⊥}^{2}\Phi \left(x,y,0\right)$$

Where z is the distance of the detector to the object, λ is the wavelength, *I*(*x*, *y*, *z*) is the detected intensity on the detector plane, and Φ(*x*, *y*, 0) is the object’s phase function. Eq. (2) establishes a linear relation between the Laplace transform of the phase function and the measured intensity data. It enables generation of contrast-outlining surfaces and structural boundaries, where the refractive index changes abruptly. The process of phase retrieval is to reformulate Eq. (2) to obtain the decrement of the real part of the object’s refractive index δ(*x*, *y*, 0) from knowledge of *I*(*x*, *y*, *z*).

The phase retrieval method we used is the error reduction algorithm [16], which consists of the following four steps: 1) Forward Fresnel transform an estimate of the complex amplitude of the object plane. 2) Replace the modulus of the resulted Fresnel transform with the measured intensity to form an estimate of the complex amplitude of the detector plane. 3) Backward Fresnel transform the estimated complex amplitude of the detector plane. 4) Constrain the amplitude to be zero out of the object zone and then form a new estimate of the complex amplitude of the object plane.

Define the complex amplitude as *U*(*x*, *y*, *z*) = *ρ*(*x*, *y*, *z*)exp[*iδ*(*x*, *y*, *z*)]. The relation between the intensity *I*(*x*, *y*, *z*) and the complex amplitude *U*(*x*, *y*, *z*) is *I*(*x*, *y*, *z*) = |*U*(*x*, *y*, *z*)|^2^.

### Respiratory phase extraction and alignment

Lung and rib cage movements are caused by a live mouse’s breathing, there are two ways to deal with this problem: one is the use of animal ventilator to control rat’s breathing rhythm; the other is anesthetized spontaneous breathing, and respiratory rhythm parameters are applied to extract the same phase of breathing images, and then rebuilt the 3D model. In this paper, we utilize the second way. The proposed alignment method is fully automatic and it includes two steps: 1) design a respiratory phase extraction algorithm that can divide the images into different phases and extract two stages. 2) aligning the stable region between each two images within the same respiration stage. So the gating information is not relying on threshold but our 2-step alignment algorithm.

In a live mouse, respiration is passive and the rib cageexpands and contracts to some degree through the action of other respiratory and accessory respiratory muscles. We took 20 images at each angle, which sampled several respiratory cycles of the live mouse.

**Figure 2:**
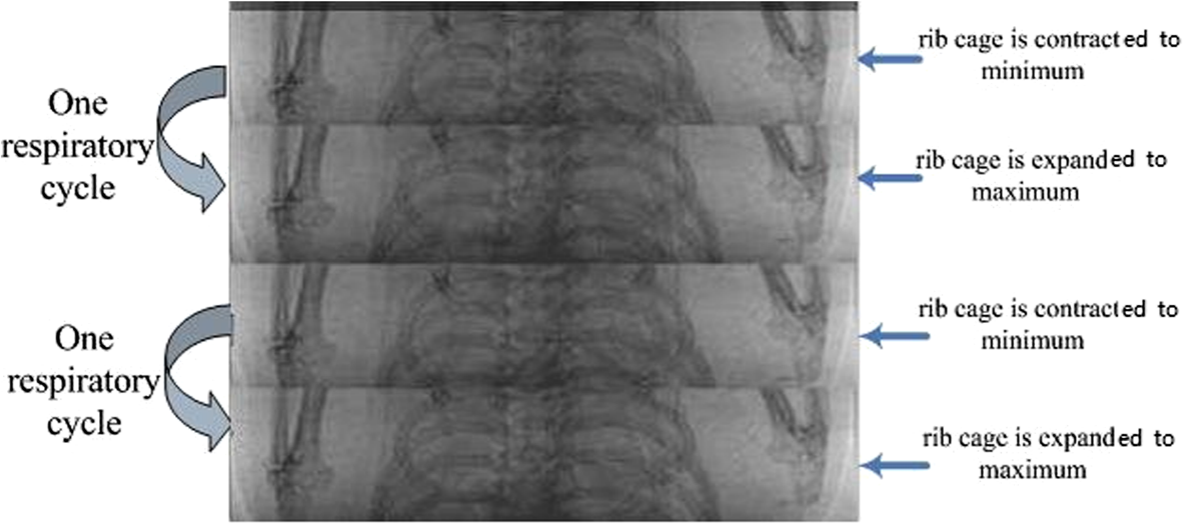
Images of two respiratory cycles viewed from one angle.

As shown in Figure 2, there are two respiratory cycles in one angle. The aim is to design a respiratory phase extraction algorithm that can divide the images into different phases and extract two stages, expanded to maximum and contracted to minimum. Two parameters, AP (average parameter) and MP (mean parameter), are calculated as

**Figure 3:**
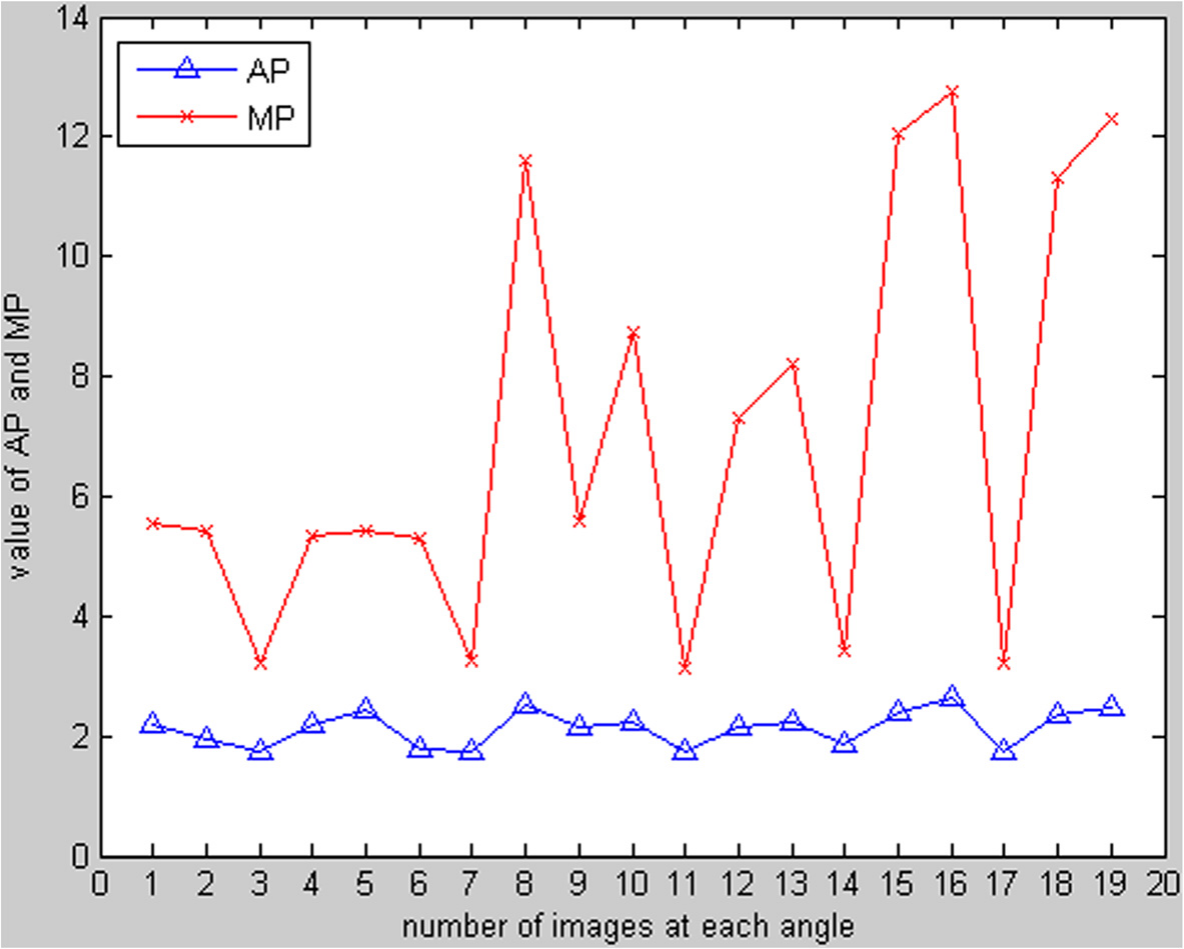
Respiratory cycles curve for one angle.

AP=∑maskIx1−Ix2Nmask

(3) $$\mathrm{MP}=\underset{\mathrm{mask}}{\text{max}}\left(∥I_{x_{1}}-I_{x_{2}}∥\right)$$

x_1_ and *x*_2_ are in a silhouette mask, which has nonzero pixels where the motion occurs. Respiratory cycle curves can be drawn after the calculation of the two parameters, as shown in Figure 3. At each angle, AP and MP are consistent, and respiratory phases are divided by AP and MP. Two stages, expand to maximum and contract to minimum, are extracted.

**Figure 4:**
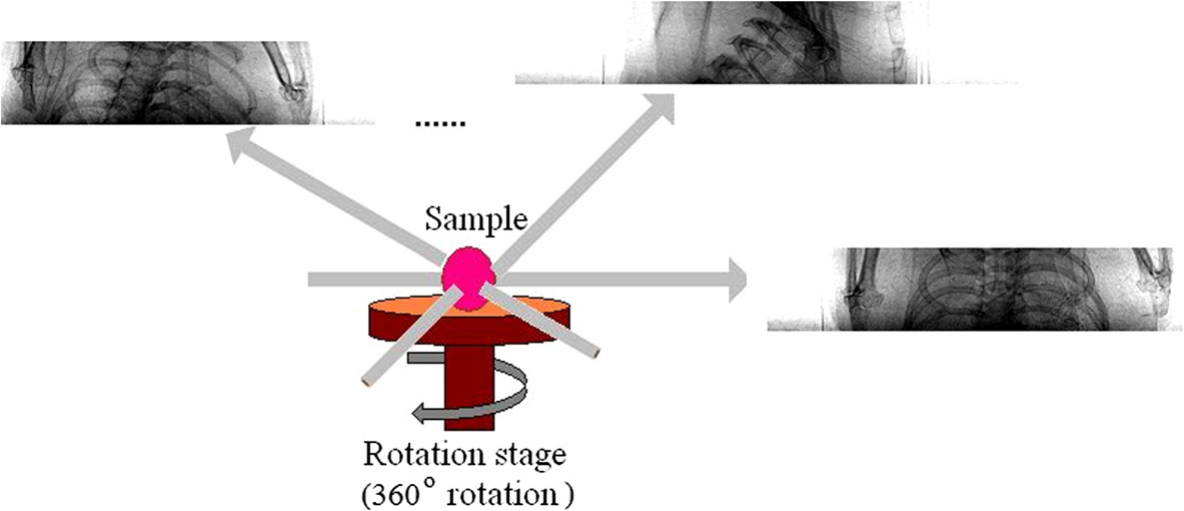
**Respiratory phase images extracted at different angles.** These images are called projection image which are taken at different angle from 1° to 180° and there are 180 images to be aligned.

Alignment of images taken at different angles is the next step. As shown in Figure 4, images taken at different angles have similar covariant characteristics if the pixels in the two images that correspond to the partial image region satisfying the following mapping:

(4) $$x\prime =H_{s}x=\left[\begin{array}{cc} \mathrm{sR}\left(\theta \right) & t\\ 0^{T} & 1 \end{array}\right]x$$

Where *x* = (*x*, *y*, 1)^*T*^ and *x*′ = (*x*′, *y*′, 1)^*T*^ denote the corresponding pixels in the two images’ homogeneous coordinates,t denotes the translation vector, *R*(θ) indicates the parameter that is a rotation matrix of the θ, and the scalar s indicates a scaling factor. The similarity transformation has four degrees of freedom (two translation parameters, a rotation parameter and a scale parameter); therefore, the shape remains after the similarity transformation.

Aligning the stable region between each two images separated by steps of 1° is important for aligning all the images together. The scheme is implemented as follows:

1) Use a 2D Gaussian kernel to continuously smooth the given gray scale image (if the input is a color image, it is necessary to pre-convert to gray scale images). Use down-sampling to construct the multi-scale representation, namely, the Gaussian pyramid. In the Gaussian pyramid for each image, by smoothing using the Gaussian kernel function, the parameter is k = 2. The adjacent images within each layer of the Gaussian pyramid are subtracted to obtain the DoG pyramids.

2) Solve for local extrema in the DoG scale space. Then, according to principal curvatures, extreme points in the instability of the position of the image edge are removed to obtain a stable (x, y, s). Here, (x, y) represents the position of the stability of extreme points and s represents the scale of extreme points.

3) Utilize the Scale Invariant Feature Transform (SIFT) feature extraction [17,18] method for each of the stable extreme points.

4) Use an exhaustive search method to solve the nearest neighbour matching relationship between two images. Then, the geometric constraints between the two image models and the RANdom SAmple Consensus (RANSAC) algorithm [19] are combined to exclude incorrect candidate matching pairs, and the geometric mapping relationship between two images is estimated.

### Compressed sensing-based CT reconstruction method

After the respiratory phase extraction and alignment scheme, images taken at different angles are aligned and prepared for reconstruction. The number is sparse: in this paper, each angle has two images (expand to maximum and contract to minimum). The reconstruction methods share the idea of compressed sensing (CS). The main premise of compressed sensing (CS) [20-22] is that although the signal is not necessarily sparse in real space or Fourier space, it is sparse or compressible in some basis. If we consider a real signal *X* = {*x*_*i*_}_1≤*i*≤*N*_ and define a real basis *Ψ* = {*ψ*_*i*,*j*_}_1 ≤ *i* ≤ *N*;1 ≤ *j* ≤ *T*_, then we can say that the decomposition α = {α_*j*_}_1≤*j*≤*T*_ is the column vector of weighting coefficients α_*j*_. The signal X can be expressed as

(5) X=Ψα

The signal X is sparse or compressible if there are only *K* < < *N* weighting coefficients that are nonzero or significant.

Suppose that each pixel is denoted by $p\left(t_{1},t_{2}\right)\begin{array}{cc} , & \left(0\leq t_{1},t_{2}<N\right) \end{array}$, and at the same position (*t*_1_, *t*_2_), the pixel in images is denoted by *q*(*t*_1_, *t*_2_). Then,

(6) $$q\left(t_{1},t_{2}\right)=\sqrt{{\left(p\left(t_{1}+1,t_{2}\right)-p\left(t_{1},t_{2}\right)\right)}^{2}+{\left(p\left(t_{1},t_{2}+1\right)-p\left(t_{1},t_{2}\right)\right)}^{2}}.$$

With any *N* × *N* digital image p, define a real number

(7) $${∥p∥}_{\mathrm{TV}}=\sum_{t_{1}=0}^{N-2}\sum_{t_{2}=0}^{N-2}\sqrt{{\left(p\left(t_{1}+1,t_{2}\right)-p\left(t_{1},t_{2}\right)\right)}^{2}+{\left(p\left(t_{1},t_{2}+1\right)-p\left(t_{1},t_{2}\right)\right)}^{2}}.$$

This is known as the image’s total variation (TV) [23]. We define that the position of (*t*_1_ + 1, *t*_2_) is ‘below’ and (*t*_1_, *t*_2_ + 1) is ‘to the right’ of (*t*_1_, *t*_2_).

We then probe the signal using M real linear measurements *Y* = {*y*_*r*_}_1≤*r*≤*M*_ in some sensing basis Φ = {*φ*_*r*,*i*_}_1 ≤ *r* ≤ *M*;1 ≤ *i* ≤ *N*_ such that

(8) Y=ΦX=ΦΨα=Θα,

where Θ = ΦΨ ∈ *R*^*M* × *T*^. The measurement process is not adaptive, which means that Ф is fixed and does not depend on the signal X. The image processing procedure consists of designing 1) a stable measurement matrix Ф and 2) a reconstruction algorithm to recover X from only M measurements Y.

The measurement matrix Ф must allow the reconstruction of the length-N signal X from M < N measurements (the vector Y). If X is K-sparse and the K locations of the nonzero coefficients in αare known, the problem can be solved with the provided M ≥ K. A necessary and sufficient condition for this simplified problem is that for any vector α_*K*_ that shares the same K nonzero entries as αand for some 0 < δ_*K*_ < 1, 

(9) $$\left(1-\delta_{K}\right){∥\alpha_{K}∥}_{2}^{2}\leq {∥\Theta \alpha_{K}∥}_{2}^{2}\leq \left(1+\delta_{K}\right){∥\alpha_{K}∥}_{2}^{2}.$$

Defining the *l*_*p*_-norm of vector u, ∥*u*∥_*p*_ = ( ∑ |*u*_*i*_|^*p*^)^1/*p*^. Usually, the locations of the K nonzero entries in αare unknown. However, a sufficient condition for a stable solution for both K-sparse and compressible signals is that Θ satisfies Eq. (7) for an arbitrary 3 K-sparse vector α_*K*_. This condition is referred to as the restricted isometry property (RIP) [24]. A related condition, referred to as incoherence, requires that the rows {φ_*r*_} of Ф cannot sparsely represent the columns {ψ_*j*_} of Ѱ, and vice versa.

The reconstruction task is as follows: suppose that X is an unknown image and that we are given M measurements Y. We must find an image *X* * that is a ‘good’ approximation of X. This *X* * has the minimal TV, defined in Eq. (6), of all those that satisfy the constraint of Eq. (7).

(10) $$\min{∥X*∥}_{\mathrm{TV}}{\begin{array}{ccc} & s.t. & ∥Y-\mathrm{\Phi X}*∥ \end{array}}_{2}\leq \epsilon .$$

The method used to minimize ∥*X* * ∥_*TV*_ is the gradient descent algorithm. The general form of the gradient descent algorithm is

(11) $$x^{\mathrm{next}}=x^{\mathrm{current}}+\beta_{\mathrm{current}}\upsilon^{\mathrm{current}},$$

where β_*current*_ is a positive real number that denotes the current step length. For a digital image X and any *k* ∊ *N*, let *s*^*k*^ ∊ ∂ ∥*X*∥_*TV*_ be a sub-gradient of ∥*X*∥_*TV*_ at *x*^*k*^ and

(12) $$\upsilon^{k}=\left\{\begin{array}{cc} -\frac{s^{k}}{∥s^{k}∥}, & \left(s^{k}\neq 0\right)\\ 0, & \left(s^{k}=0\right) \end{array}\right).$$

We now describe the iterative steps of this reconstruction algorithm for phase sensitive CT images. Denote the digital image estimate by *X*^*k*^, its ith pixel by *x*_*i*_^*k*^, the measurement by Y, and the measurement matrix by Ф. The algorithm is performed according to the following pseudo code:

1. *X*^0^ = 0; β = 1; 

2. $x_{i}^{1}=x_{i}^{0}+\lambda \cdot \phi_{r,i}\cdot \frac{Y_{r}-\sum_{i=1}^{N}\phi_{r,i}\cdot x_{i}^{0}}{\sum_{i=1}^{N}\phi_{r,i}^{2}}$

3. for $k=\begin{array}{ccc} 1, & \cdots , & K \end{array}$

4. if (∥*X*^*k*^∥_*TV*_ < ∥*X*^*k*−1^∥_*TV*_)

5. *x*_*i*_^*k*^ = *x*_*i*_^*k*^ + βυ^*k*^; 

6. $x_{i}^{k+1}=x_{i}^{k}+\lambda \cdot \phi_{r,i}\cdot \frac{Y_{r}-\sum_{i=1}^{N}\phi_{r,i}\cdot x_{i}^{k}}{\sum_{i=1}^{N}\phi_{r,i}^{2}};$

7. if $\left(\sum_{i}\left|x_{i}^{k+1}-x_{i}^{k}\right|<\epsilon \right)$

8. break;

9. else

10. β = 0.8β; 

11. end if

12. else

13. β = 0.8β; 

14. * x*_*i*_^*k*^ = *x*_*i*_^*k*^ + βυ^*k*^

15. end if

ϵ is a small positive value to control the iterative process. To avoid infinite loops, we also introduced an additional stopping criterion based on the maximum loop iterations, K. When the iterative process is stopped, the phase retrieval process is begun. These parameter values are experimentally set, β = 0.8β is experimentally tested. In our experiment, gradient descent can start the process of decline more rapidly, as extreme approach, the rate of decline to slow down to make sure it converges to the optimum. λ is in the range [0, 2]. It is called relaxation factor, which is affecting convergence speed and reconstruction quality. There is a large number of specialized research focus on this topic. In this paper, relaxation factor values is in [0, 2] range. The bigger of Relaxation factor, the faster iteration convergence, but the result is more unstable. The smaller of Relaxation factor, the slower iteration convergence, but can guarantee convergence to a better optimum.

## Results

### Experiment implementation

**Figure 5:**
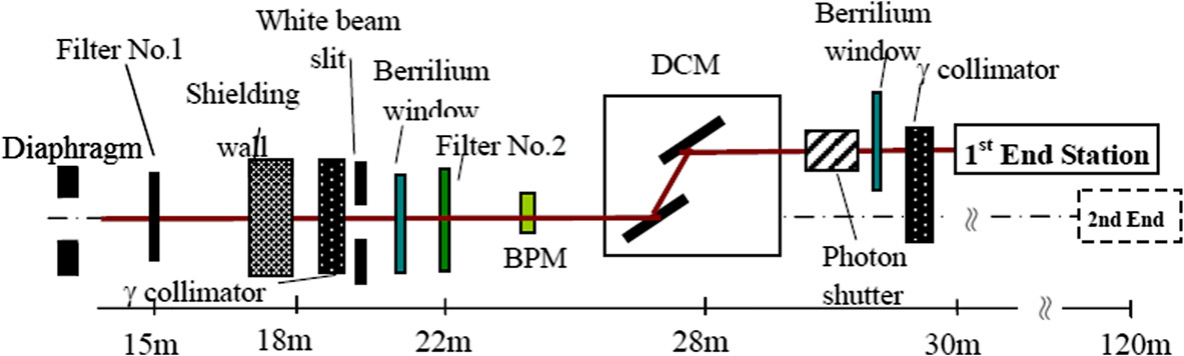
Schematic of the BL13W1 beamline at the SSRF.

The experiments were performed at the BL13W1 beamline at the Shanghai Synchrotron Radiation Facility (SSRF) in China. The light source was a hybrid-type wiggler with periodic length of 14 cm and period number 8. The radiating power was varied from 8.0 to 72.5 keV by tuning the gap from 17 mm to 35 mm. A fixed-exit double-crystal cryogenic-cooling monochromator was placed 28 m away from the source. The monochromator crystal was a combination of an Si(111) orientation crystal and an Si(311) orientation crystal. A high-precision sample platform was used to position the sample and rotate the sample axis perpendicular to the beam. The space resolution of the sample platform was better than 1 micron. Figure 5 shows a schematic of the BL13W1 beamline at the SSRF [24].

The specimen was a 4-week-old live mouse. It was fixed to the sample rotation platform. The edge-enhancement images were measured at 180 different angular settings of the specimen. The white beam X-ray (wide band photoelectron spectroscopy X-ray) was monochromatized at 25 keV using a silicon double-crystal monochromator. The distance from the sample to the detector could be as great as 1.2 m, which would achieve the greatest phase shift and the best image in this experiment. The sample was placed on a high-precision sample platform and was scanned rotationally at 1° per step over 180°. The exposure time was 24 ms for each scanned step. The transmitted radiation was detected by the X-ray CCD (13 μm/pixel). All experiments and procedures carried out on the animals were approved by the animal welfare committee of Capital Medical University and the approval ID is SCXK-(Army) 2007–004.

### Respiratory phase extraction and alignment

**Figure 6:**
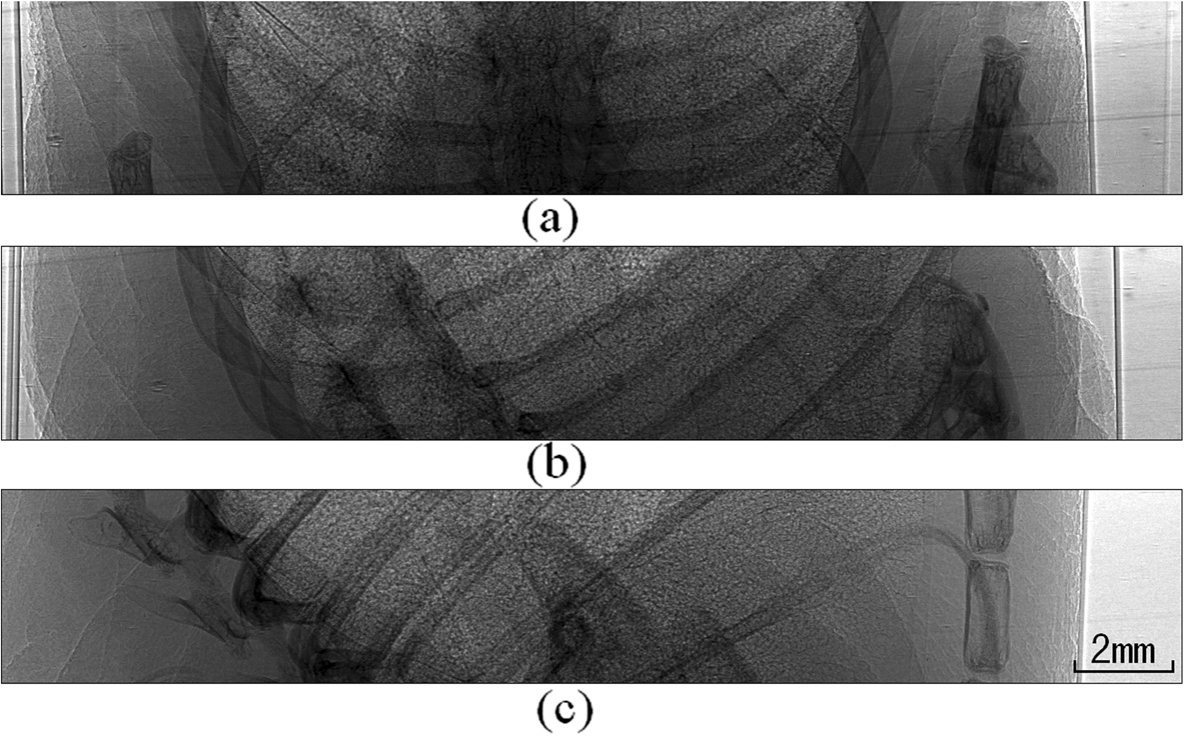
Alignment results for two images at different angles, 0° and 1.

Respiratory cycle curves can be drawn after the calculation of the two parameters. Two stages, expand to maximum and contract to minimum, are extracted. Alignment of images taken at different angles is the next step (Results shown in Figure 6). Images taken at different angles have similar covariant characteristics. In this paper, 180 images from 1° to 180° are aligned. The performance of alignment algorithm is listed in Table 1. Here we take 1° to 5° for example to illustrate the numerical results of time and number of matching points.

**Table 1:** Alignment results

**Series number**	**Candidate in image #1**	**Candidate in image #2**	**Number of matched points**	**Time cost for matching(sec)**
1° to 2°	85	160	54	0.126
2° to 3°	160	90	52	0.126
3° to 4°	90	125	59	0.128
4° to 5°	125	118	54	0.136
1° to 3°	85	90	32	0.120
3° to 5°	90	118	30	0.123
1° to 5°	85	118	5	0.123

Performance results (number of matching points and time cost) of alignment from the projection angle 1° 2° 3° 4° 5°.

### Reconstruction results of in vivo mouse lung

**Figure 7:**
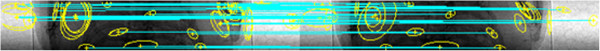
Three in-line phase-contrast experimental projection images of the mouse’s chest taken from several different angles.

**Figure 8:**
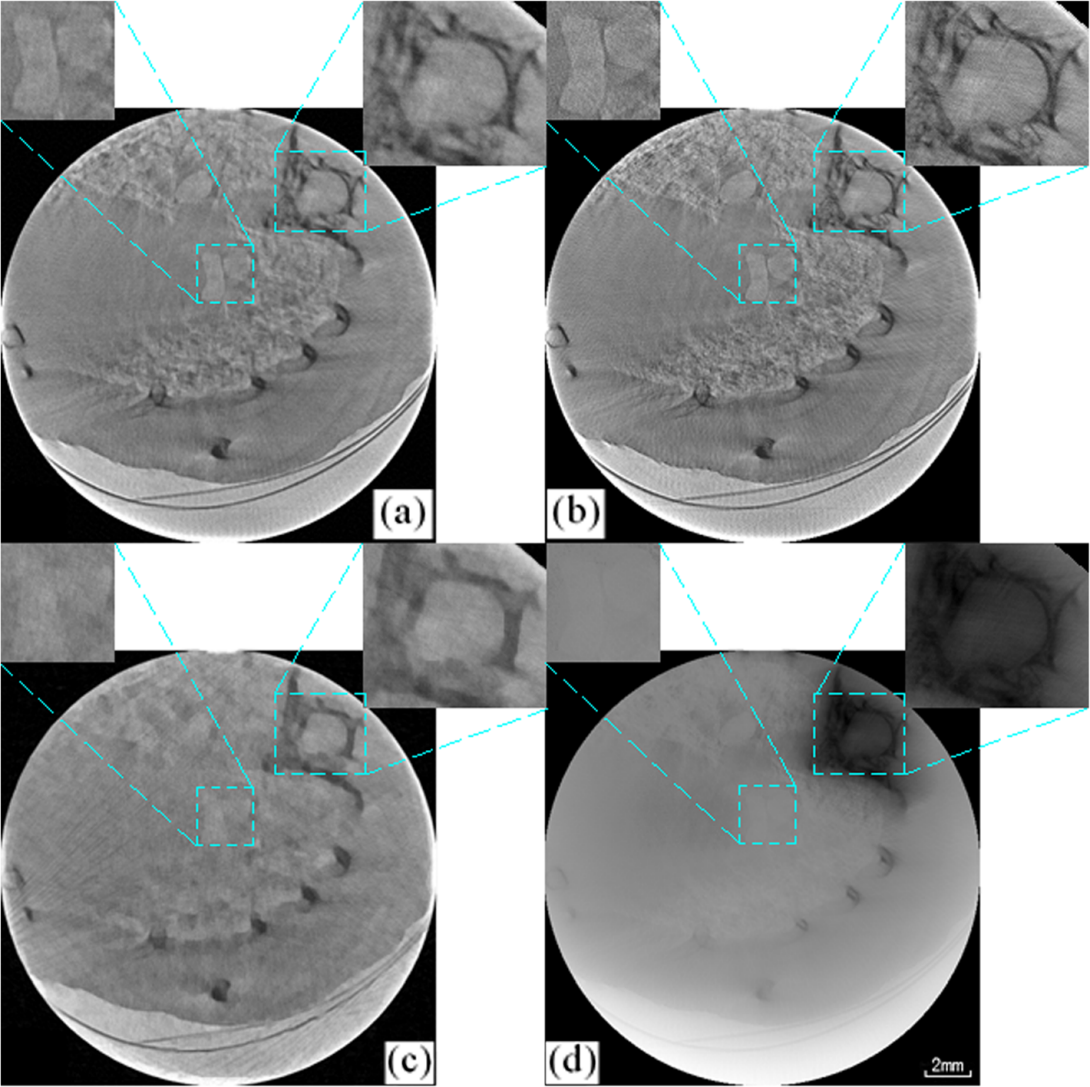
**Alignment results for two images at different angles, 0° and 1.** The CT reconstructed image of the 70th slice using **(a)** the CS-based method from 60 projection images, **(b)** the CS-based method from 180 projection images, **(c)** the CS-based method from 30 projection images, and **(d)** the FBP method from 180 projection images.

Figure 7 shows three in-line phase-contrast projection images of the mouse’s chest taken at different angles. Figure 8 shows the CT reconstructed images for one slice reconstructed using two algorithms under three sampling conditions. All the images come from the same slice, and the total number of slices is 105. The images are (a) the CT image reconstructed via the CS-based algorithm from 60 views, (b) the CT image reconstructed via the CS-based algorithm from 180 views, (c) the CT image reconstructed via the CS-based algorithm from 30 views, and (d) the CT image reconstructed via the FBP algorithm from 180 views. From the results, we can see that the CT images reconstructed via the CS-based algorithm have better quality than the CT images from the FBP algorithm. Even the CS-based method with less views, such as 30, has better performance than the FBP method with more views, such as 180. This result implies that the proposed novel CS-based CT reconstruction method could be applied with a lower exposure time and dose. Our algorithm could address the respiration of a live mouse and reconstruct the 3D rib cage using very sparse image data.

## Conclusions

A novel compressed sensing (CS) theory-based CT reconstruction method was proposed, and an experiment was performed to test the breathing phase extraction method as applied to the lung and rib cage movement of a live mouse. The results demonstrate that our method can reconstruct the images using less sampling data than FBP while maintaining the same high quality.

## Discussion

Several topics must be further studied and discussed:

1) The alignment of images taken at different angles requires further study. This paper could address the respiration of a live mouse by respiratory phase extraction. However, lung tissue reconstruction requires further study on the alignment topic because a more accurate alignment algorithm would result in more robust 3D reconstruction of lung tissue.

2) The algorithm remains time-consuming, which presents a difficulty for clinical applications. Methods for designing a fast and real-time in-line phase-contrast X-ray CT system are important and require further research.

## Abbreviations

ILPCI: In-line phase-contrast imaging; CT: Computed tomography; FBP: Filtered back projection; CS: Compressed sensing; PCI: Phase-Contrast imaging; DEI: Diffraction-enhanced imaging; ESRF: European synchrotron radiation facility; CSIRO: Commonwealth scientific and industrial research organization; PCX-CT: Phase contrast x-ray computed tomography; TIE: Transport of intensity equation; SIFT: Scale invariant feature transform; RANSAC: RANdom sample consensus; SSRF: Shanghai synchrotron radiation facility.

## Competing interests

The authors declare that they have no competing interests.

## Authors’ contributions

LW carried out the respiratory phase extraction and alignment studies, participated in the testing of algorithm performance and drafted the manuscript. XL carried out the CS reconstruction algorithm and implementation. MW performed the statistical analysis. SL conceived of the study, and participated in its design and coordination and helped to draft the manuscript. All authors read and approved the final manuscript.
